# Self-perceived job insecurity and self-reported health: Differences between native-born and migrant workers based on evidence from the Sixth European Working Conditions Survey

**DOI:** 10.1371/journal.pone.0267252

**Published:** 2022-04-29

**Authors:** Nunzia Nappo

**Affiliations:** Department of Political Science, Università di Napoli “Federico II”, Napoli, Italia; University of Rome, ITALY

## Abstract

This paper analyses the association between self-perceived job insecurity and self-reported health by comparing two population groups, native-born and migrant workers, in EU15 countries. The econometric analysis employs data from the Sixth European Working Conditions Survey that was released in 2017. The health outcome examined in this study is self-reported health, which is a subjective indicator. Self-perceived job insecurity is an individual’s subjective evaluation of the possibility of future job loss. The association between job insecurity and self-reported health was tested using standard probit models and standard ordered probit models, considering the entire population sample, only native-born workers, only migrant workers. The results show that workers who think that they might lose their jobs have a lower probability of reporting very good and good health than workers who do not worry about losing their jobs, with job insecurity reducing the probability of reporting good health more for migrant workers than for native-born workers.

## Introduction

One result of increasing globalization in recent decades is that most workplaces have experienced restructuring and reorganization to ensure competitiveness. Downsizing and the introduction of labour-saving technologies have affected many organizations. In addition, the share of temporary and part-time work has been increasing [1]. These and other changes in working conditions have impacted workers’ concerns about losing their jobs [2, 3], which has had negative consequences for the health of these employees [4–7]. According to Eurofound and the International Labour Organization [8], “job insecurity is of concern across many countries, with one out of six workers in the EU and one out of every ten workers in the US worried that they might lose their job in the next six months”. The aim of this paper is to investigate whether self-perceived job insecurity is associated with self-reported health and whether the association differs between two population groups, i.e., native-born and migrant workers, in EU15 countries. Health is considered a multidimensional outcome influenced by 1) standard workers’ sociodemographic characteristics, 2) certain job features, such as job insecurity, and 3) individual lifestyles. The empirical analysis employs data from the Sixth European Working Conditions Survey (EWCS6) that was released in 2017 [9].

According to Greenhalgh and Rosenblatt [10], job insecurity is “the perceived powerlessness to maintain desired continuity in a threatened job situation”. van Vuuren and Klandermans [11] define job insecurity as workers’ “concern about the future permanence of the job”. Based on the above definitions, self-perceived job insecurity can be described as the worrisome thoughts workers have about the future stability of their jobs. Job insecurity is a subjective experience that can be the result of an objective threat (such as the pending downsizing of a plant) too. Self-perceived job insecurity can differ between co-workers who share the same objective circumstances [1]. Whether or not and the degree to which workers are concerned about the probability of losing their jobs depend both on the macroeconomic situation and on the extent to which each worker feels confident about the possibility of finding a new job. Job insecurity can vary across workers with different social characteristics and among population groups. Workers who are most exposed to job insecurity are those with low education levels who have been previously unemployed [12] and migrants who are employed in jobs with poor working conditions, including job insecurity [13], far more frequently than native-born workers [14].

Job insecurity has several consequences at both the individual and organizational levels [15]. Workers who feel they are in precarious employment situations are likely to have reduced productivity, which increases costs for their employers. For workers, the persistence of the perceived insecurity is likely to become stressful. “Job insecurity can be one of the more important stressors in employment situations” [16], with implications for workers’ wellbeing [17]. These are the main reasons studying job insecurity is important, and the reduction of job insecurity should be a priority for both national governments and the international institutions.

The perceived threat of unemployment has consequences for several health indicators: job insecurity impacts psychological and physical morbidity, mortality, sickness-related absences and health care utilization [18]. However, a large body of literature (see, for instance, [19, 20]) has shown that the negative effects of job insecurity on health are influenced by the duration of the experience: the longer the concern about the loss of their jobs persists, the worse the health outcomes are for the impacted workers. In addition, it seems that the effects of job insecurity on health are not rapidly reversed by a worker finding a secure job [21].

Lubke [12] suggests at least four possible ways in which job insecurity could impact health: 1) workers, because they are concerned about job loss, live in a precarious state that is likely to produce stress reactions, which, in turn, affect their health; 2) the perception of the risk of losing their jobs is likely to make workers perceive their financial situations as being more precarious, which adds stress that further negatively affects their health outcomes; 3) job insecurity can result in a perception of having a lack of control over life circumstances in general, and people who perceive that they have adequate personal control enjoy better health than people with a reduced sense of control. And 4) job insecurity can impact health indirectly through a reduction in family satisfaction, as people who are worried about job instability are more likely to transfer their job insecurity stress onto their family. According to Caroli and Godard [6], another reason job insecurity has detrimental effects on health could be that workers who are worrying about their job stability could choose to increase the amount of money they are saving by reducing investments in their own health.

One of the problems with the literature on the relationship between job insecurity and poor self-reported health is reverse causality. Most studies provide results regarding the correlations between health and job insecurity without estimating the causal effect of job insecurity on health. It could be that unhealthy people are more likely to be employed in less secure jobs or, on the contrary, that adverse health events make people more likely to worry about the probability of losing their jobs. In addition, most research has focused on single countries and/or on advanced wealthy countries for which data are available. There are very few cross-national analyses that address the direction of the link between self-reported health and job insecurity. One study that does is László et al. [22]. The authors studied the association between job insecurity and self-rated health by analysing a sample of 45- to 70-year-old workers from 16 European countries. The results show that job insecurity is significantly associated with an increased risk of poor health in 9 of the 16 nations. Caroli and Godard [6] assess the causal effect of perceived job insecurity on health in a sample of men from 22 European countries. The findings indicate that when the potential endogeneity of job insecurity is not accounted for, there are negative impacts on almost all health outcomes [6].

The literature on the relationship between job insecurity and self-reported health has been growing over time; however, scant attention has been given to differences among native-born workers and migrant workers, especially within Europe. Data show that migrants are often employed in precarious or insecure jobs [23] and have a higher level of job insecurity than native-born workers [24]. Daly et al. [25] report that higher psychological distress among migrant workers in Australia was associated with factors such as jobs with low security. According to Liu et al. [26], reducing migrants’ job insecurity could reduce occupational mental health inequities for migrant workers in Australia. In a review of the relevant studies on the role of work organization and occupational health disparities, Landsbergis et al. [23] found stronger associations between job insecurity and health among migrant workers than among native-born workers. Hasan et al. [27] provides a review of the evidence for the prevalence of depression and anxiety among migrant workers by investigating the risk factors and the availability of social support for migrant workers. The review shows an association between occupational stressors (including job insecurity) and mental health issues among migrant workers. Migrant workers report a worse level of mental well-being due to the presence of several job characteristics, including high job insecurity. Daly et al. [28] found that in Australia, the impact of vulnerability and insecurity on overall health indicators differs by migration status with native-born workers self-reporting poorer health, than workers born elsewhere.

To the best of our knowledge, very little is known about the impact that job insecurity has on the health of migrant workers in the EU. This study aims to expand the literature on the association between job insecurity and self-reported health by analysing differences among native-born and migrant workers within EU15 countries. Migrants are identified as a more “vulnerable group” with regard to job insecurity than native-born workers. To the best of our knowledge, this issue has not been addressed for EU15 countries. This represents the original contribution of the paper to the literature. In addition, this is the first time that the EWCS6 has been employed with this aim. The main weakness of the paper is the assessment of the association between job insecurity and self-reported health without defining the direction of the causal link between the two elements.

The paper is organized as follows. The next section explains the data and the model, followed by a section including the results and a discussion of them, and finally a concluding summary.

## Materials and methods

The econometric analysis employs data from the Sixth European Working Conditions Survey. The data were accessed and downloaded via the UK Data Service (dataset name 10.5255/UKDA-SN-8098-4). The terms of service for the website from which the data were collected were complied with. Data from the EWCS6 were collected in 2015 and released in 2017. Although quite old, the survey is the latest available and data collected provide one of the most accurate and reliable pictures of working conditions with regard to migrant workers at European level. Eurofound [9] provides a complete description of the survey design. The survey offers an exhaustive picture of work in Europe over time and across countries, occupations, genders and age groups and presents an overview of the working conditions in Europe. Approximately 43,000 casually selected workers age 15 or over were interviewed face-to-face. The questionnaire includes questions related to employment status, working time schedules, work organization, workers’ training, physical and psychosocial risk factors, work environment, health and safety, work-life balance, earnings, and financial security. The whole sample consists of 35 countries, including the EU28 plus Norway, Switzerland, Albania, the former Yugoslav Republic of Macedonia, Montenegro, Serbia, and Turkey. Unfortunately, a panel dimension of the data is not available. The econometric analysis focuses on the EU15: Austria, Belgium, Denmark, Finland, France, Germany, Greece, Ireland, Italy, Luxembourg, Netherlands, Portugal, Spain, Sweden, and the United Kingdom. The sample includes both employed and self-employed workers. After removing the unselected respondents and those with missing data for the dependent and independent variables, the final complete dataset consists of 18,071 observations. With the aim of comparing the correlation between job insecurity and health among native-born and migrant workers, the dataset has been split into two subsamples, one with native-born workers, consisting of 14,995 observations, and one with migrant workers, consisting of 3,076 observations.

### Dependent variable

The dependent variable is self-reported health (*SRH*), which is a subjective indicator. *SRH* can be considered a reliable indicator of general health [29]. Indeed, self-reported health seems to be significantly correlated with doctors’ evaluations of health and is a good predictor of morbidity and mortality [30]. In addition, it is not clear if more objective health indicators are completely free from reporting error. For this reason, although more objective measures of health are becoming more available, *SRH* can still be considered a good proxy for health [31].

*SRH* was collected through individual interviews. Interviewees were asked, “How is your health in general? Would you say it is….?”. Answers were expressed on a scale of values from one (*very good*) to five (*very bad*). In the literature (see, among others, [22]), *SRH* is frequently considered a binary variable. Responses have been grouped into two categories: the first category “*good health*” includes ‘very good’ and ‘good’ answers, while the second category “*bad health*” includes ‘fair’, ‘bad’ and ‘very bad’ answers. In addition, *SRH* has been considered a four values variable from one (*very good health*) to four (*bad* and *very bad health*). Table 1, which reports the characteristics of the study sample, shows the descriptive statistics for the dependent variable by country and by population group. Data show that the percentage of workers who evaluate their health as “*good health*” is almost the same in both population groups and is equal to 79.2% of native-born workers and 79.5% of migrant workers. However, there are substantial differences within the EU15, with some countries in which the percentage of native-born workers reporting good health is much higher than the percentage of migrant workers. Examples include Austria (80.1% versus 71%), France (82.6% versus 75.2%) and the Netherlands (82.9% versus 77.2%). In contrast, there are some countries in which the percentage of native-born workers reporting “good health” is much lower than the percentage of migrant workers. This is the case in Portugal (67.7% versus 86.9%) and Italy (53.3% versus 66.6%). There are only two countries, Belgium and Germany, where the percentage of native-born and migrant workers reporting good health is almost the same.

**Table 1:** Characteristics of the study populations.

Country	N	Natives (%)	Natives Job Ins (%)	Migrants Job Ins (%)	Self-rated health (%) Natives					Self-rated health (%) Migrants				
					*Very good*	*Good*	*Fair*	*Bad*	*Very bad*	*Very good*	*Good*	*Fair*	*Bad*	*Very bad*
Austria	959	84.8	9.2	16.3	35.8	44.3	17.2	2.0	0.4	33.1	37.9	23.4	4.8	0.6
Belgium	2,379	80.0	13.1	19.3	27.0	53.5	17.1	1.8	0.3	29.6	50.5	16.8	2.5	0.4
Denmark	886	88.3	8.7	7.2	41.6	42.4	14.1	1.4	0.3	38.8	42.7	15.5	2.9	0.0
Finland	929	98.0	15.5	23.5	21.3	56.4	20.4	1.6	0.2	16.6	55.5	22.2	5.5	0.0
France	1,478	75.6	13.3	17.0	24.0	58.6	15.4	1.6	0.2	26.6	48.6	21.6	2.7	0.2
Germany	1,888	75.6	10.1	12.7	18.8	55.4	23.5	1.8	0.2	18.3	56.6	20.4	4.5	0.0
Greece	974	91.7	20.8	29.8	48.1	43.4	7.9	0.4	0.1	58.7	35.0	5.0	1.2	0.0
Ireland	1,038	82.2	15.2	17.4	48.2	42.9	7.8	0.7	0.0	47.2	39.6	11.4	1.6	0.0
Italy	1,290	93.9	19.1	47.6	14.0	52.3	29.7	3.3	0.4	17.9	48.7	29.4	3.8	0.0
Luxembourg	967	33.4	9.6	10.5	20.7	60.3	15.4	3.1	0.3	25.1	51.8	20.3	1.7	0.6
Netherlands	962	83.4	24.2	21.5	27.7	55.2	14.4	1.6	0.5	23.2	54.0	18.8	3.7	0.0
Portugal	899	93.9	17.2	37.7	14.5	53.2	27.8	3.6	0.5	24.0	62.9	11.1	1.8	0.0
Spain	3,284	87.5	24.5	36.1	23.7	54.0	19.2	2.6	0.2	24.2	56.7	16.1	2.2	0.4
Sweden	957	79.0	12.1	15.3	28.1	51.8	16.5	2.9	0.5	35.0	47.5	14.5	3.0	0.0
UK	1,585	76.3	12.6	15.0	34.4	47.1	16.4	1.8	0.0	33.4	51.0	14.1	0.5	0.2
EU15	20,475	83.0	15.8	18.8	27.3	51.9	18.3	2.0	0.3	29.3	50.2	17.7	2.4	0.3

### Independent variables

Individual health depends on a plurality of factors the complex interactions of which produce distinct states of health. Those factors, which have different natures (social, demographic, educational, financial, psychosocial, environmental), can be correlated with the individuals’ perceived health status [32]. In this paper, the independent variable of interest is self-perceived job insecurity, which is the individual’s subjective evaluation of the possibility of future job loss. The EWCS6 assesses job insecurity with the following question: “To what extent do you agree or disagree with the following statements about your job? I might lose my job in the next 6 months” (Question 89g). Respondents could express their answers using a five-point scale from “*strongly agree*” to “*strongly disagree*”. Answers are grouped by aggregating (1) “strongly agree” and “tend to agree” and (2) “neither agree nor disagree”, “tend to disagree” and “strongly disagree”. Therefore, job insecurity is studied as a dummy variable (*Job insecurity*) that equals 1 if the individual thinks that they might lose the job in the next 6 months and 0 otherwise. Table 1 shows that the percentage of workers reporting job insecurity is higher among migrant workers than among native-born workers (18.8% versus 15.8%). This is true for all countries, with the exception of Denmark and the Netherlands, where the percentage of workers reporting job insecurity is higher among native-born workers than among migrant workers. As Table 1 shows, there are some countries where the percentage of migrant workers’ who perceive their jobs to be insecure is much higher than that of native-born workers. In Italy, the percentage of migrant workers who think that they might lose their jobs is 47.6%, while the percentage of native-born workers is 19.1%. Countries where the difference in perceived job insecurity between the two population groups is the highest include Portugal, Spain, Greece, and Finland. In contrast, the countries where this difference is the lowest are Luxemburg, Ireland, and Germany.

The other key independent variable is the status of the migrant worker. Individuals were asked if they and both of their parents were born in the country in which they work. Workers who responded “YES” are identified as native-born workers. They constitute 83.0% of the sample. Workers who responded “NO” are identified as migrant workers. Therefore, the negative answer to the question (not born in the EU and/or born in the EU but not born to parents born in the EU) is considered a proxy for migrant status. The migrant group is composed of workers who were born in the country to parents who were not born in the country (28.11% of the sample, which equals 978 workers) and workers who were not born in the country to parents who were also not born in the country (71.80% of the sample or 2,498 workers).

The model includes standard sociodemographic variables. Table 2 provides the definitions of the independent variables. Age was classified into five groups. Gender is a dummy variable. Individuals were classified according to marital status as having a partner or being single. Educational attainment was classified into three levels based on the highest level of education attained: primary education, secondary education, and tertiary education (used as the reference group). Occupational status was categorized as managerial versus nonmanagerial. The number of hours usually worked per week in the workers’ main paid job was included. It was also considered whether workers were employed part-time (on average up to 6 h of work/day) or full-time (on average 6–8 hours of work/day), the standard definition of full-time work includes 35 h per week or more and less than 35 h per week for part-time workers. The number of hours that individuals spent in sport, cultural or leisure activities performed outside their home and their difficulty falling asleep were controlled for in the analysis. It was considered if workers had health problems over the last twelve months: if they suffered from anxiety and if they suffered from overall fatigue. Fourteen country dummies were included in the models, considering the UK as the reference country.

**Table 2:** Definition of the independent variables.

*Variable*	*Description*
Age1	1 if she/he is 15/25 years old, 0 otherwise
Age2	1 if she/he is 16/26 years old, 0 otherwise
Age3	1 if she/he is 37/47 years old, 0 otherwise
Age4	1 if she/he is 48/58 years old, 0 otherwise
Age5	1 if she/he is 59/89 years old, 0 otherwise (reference group)
Male	1 if male, 0 otherwise
Native	1 if she/he and both of her/his parents were born in the Country, 0 otherwise
Partner	1 if she/he has a spouse or a partner, 0 otherwise
Low Education	1 if highest level of education is primary education, 0 otherwise
Middle Education	1 if highest level of education is secondary education, 0 otherwise
High Education	1 if highest level of education is tertiary education, 0 otherwise (reference group)
Manager	1 if she/he is a manager, 0 otherwise
Workedhours	N. of hours usually worked per week in the main paid job
Partime	1 if she/he works part time, 0 otherwise
Jobinsecurity	1 if she/he thinks that might lose the job in the next 6 months, 0 otherwise
Sport	N. of hours she/he practices sport, cultural or leisure activities outside her/his home
Badsleep	1 if she/he has difficulty falling asleep, 0 otherwise
Anxiety	1 if she/he suffered from anxiety over the last 12 months, 0 otherwise
Fatigue	1 if she/he suffered from fatigue over the last 12 months, 0 otherwise
Austria	1 if the country is Austria, 0 otherwise
Belgium	1 if the country is Belgium, 0 otherwise
Denmark	1 if the country is Denmark, 0 otherwise
Finland	1 if the country is Finland, 0 otherwise
France	1 if the country is France, 0 otherwise
Germany	1 if the country is Germany, 0 otherwise
Greece	1 if the country is Greece, 0 otherwise
Ireland	1 if the country is Ireland, 0 otherwise
Italy	1 if the country is Italy, 0 otherwise
Luxembourg	1 if the country is Luxembourg, 0 otherwise
Netherlands	1 if the country is Netherlands, 0 otherwise
Portugal	1 if the country is Portugal, 0 otherwise
Spain	1 if the country is Spain, 0 otherwise
Sweden	1 if the country is Sweden, 0 otherwise
UK	1 if the country is the UK, 0 otherwise (reference country)

### Econometric models

The association between job insecurity and self-reported health was tested using standard probit models and standard ordered probit models. Regarding the former, three models have been examined. The first model includes all of the population samples with the selected characteristics. The second model considers only native-born workers. The third model considers only migrant workers. Outcomes of the probit model describe a correlation rather than a cause-and-effect relation between job insecurity and self-reported health. Marginal effects, which measure the expected instantaneous change in the dependent variable as a function of a change in a certain explanatory variable while keeping all other covariates constant, have been calculated for the three models. Marginal effects allow us to interpret the effect of the regressors on the dependent variable. Regarding the ordered probit models, the marginal effects of the regressors, expressed in terms of a change in the independent variables on the probability of reporting “*very good health*” and on the probability of reporting “*bad* and *very bad health*” have been assessed. Marginal effects give information on the magnitude of the correlations between self-reported health and job insecurity. Four models have been examined. The first and the third model (the probability of reporting “*very good health*” and the probability of reporting “*bad* and *very bad health*” respectively) consider only migrant workers (see Table 4). The second and the fourth model (the probability of reporting “*very good health*” and the probability of reporting “*bad* and *very bad health*” respectively) consider only native-born workers (see Table 4).

## Results

Table 3 shows the marginal effects of a change in regressors on the probability of reporting good health for the three probit models (the entire population, the native-born workers, and the migrant workers). Marginal effects assess the expected change in the dependent variable as a function of a change in a specific explanatory variable, while all other covariates hold constant. In a probit model, interpreting marginal effects is problematic because they are not equal to the coefficients; moreover, their signs are not necessarily identical to the signs of the coefficients [33]. However, marginal effects provide insight into the extent of the correlation between self-reported health and the covariates. Looking at Table 3, it appears that marginal effects, i.e., the extent of the correlation between health and the independent variables, are quite similar for both population groups, with the significant exception of self-perceived job insecurity. Regarding the first model (the total population), workers who think that they might lose their jobs in the next 6 months have a lower probability of reporting good health than workers who do not think they may lose their jobs in the next 6 months. Looking at the two population groups, both native-born and migrant workers, the perception of job insecurity decreases the probability of reporting good health; however, job insecurity reduces the probability of reporting good health more for migrant workers than for native-born workers. Younger workers have a higher probability of reporting good health than older workers. Males have a 1% lower probability of reporting good health than females. Native-born workers have a 1% higher probability of reporting good health than migrant workers. Workers who live with a partner have a 2%higher probability of reporting good health than workers who live alone. Workers with lower education levels have a lower probability of reporting good health than workers with higher education levels. The lower the level of educational attainment is, the lower the probability of reporting good health. Managers have a lower probability of reporting good health than workers who have a nonmanagerial occupational status. Part-time workers have a lower probability of reporting good health than full-time workers. As the hours spent practising sports and enjoying cultural or leisure activities outside the home increase, the probability of reporting good health increases. Workers who have difficulty falling asleep have a 13% lower probability of reporting good health than workers who do not have such difficulty. Compared to UK workers (the UK was considered the reference country), workers from Austria, Belgium, Finland, Germany, Italy, Luxemburg, Portugal, and Spain have a lower probability of reporting good health. In contrast, compared to UK workers, workers from both Greece and Ireland have a higher probability of reporting good health.

**Table 3:** Marginal effects. *SRH* as binary variable.

*Variable*	*All the sample*		*Natives*		*II gen migrants*	
	*dx/dy*	*P > | z |*	*dx/dy*	*P > | z |*	*dx/dy*	*P > | z |*
Age1	.14	0.000***	.14	0.000***	.12	0.000***
Age2	.13	0.000***	.14	0.000***	.12	0.000***
Age3	.10	0.000***	.10	0.000***	.08	0.001***
Age4	.05	0.000***	.05	0.000***	.05	0.052*
Male	-.01	0.010***	-.01	0.017**	-.01	0.375
Native	.01	0.076*				
Partner	.02	0.001***	.02	0.002***	.02	0.176
Low Education	-.07	0.000***	-.07	0.000***	-.07	0.005***
Middle Education	-.03	0.000***	-.03	0.001***	-.03	0.073*
Manager	-.02	0.001***	-.02	0.003***	-.03	0.094*
Partime	-.02	0.000***	-.02	0.000***	-.01	0.396
Jobinsecurity	-.04	0.000***	-.04	0.000***	-.07	0.000***
Sport	.07	0.000***	.07	0.000***	.07	0.000***
Badsleep	-.13	0.000***	-.13	0.000***	-.13	0.000***
Austria	-.04	0.014**	-.02	0.180	-.15	0.005***
Belgium	-.03	0.028**	-.02	0.076*	-.04	0.211
Denmark	.01	0.417	.02	0.251	-.02	0.676
Finland	-.04	0.026**	-.03	0.084*	-.13	0.264
France	-.01	0.419	.00	0.795	-.06	0.056*
Germany	-.05	0.001***	-.04	0.011**	-.07	0.085*
Greece	.11	0.000***	.11	0.000***	.14	0.000***
Ireland	.08	0.000***	.09	0.000***	.01	0.709
Italy	-.14	0.000***	-.13	0.000***	-.17	0.014**
Luxembourg	-.04	0.018**	-.01	0.523	-.08	0.011**
Netherlands	-.02	0.171	-.01	0.375	-.05	0.201
Portugal	-.07	0.001***	-.07	0.001***	.02	0.746
Spain	-.03	0.007***	-.03	0.014**	-.016	0.626
Sweden	-.01	0.369	-.01	0.422	-.016	0.672

*** stat. signif. at 1%;

** stat. signif. at 5%;

* stat. signif. at 10%.

Table 4 shows the marginal effects of the ordered probit models, which assess *SRH* as four values variable, with one being “*very good SRH*” and four “*bad* and *very bad SRH*”. For a better specification, the ordered probit models contain independent variables included in the probit model and *Workedhours*, *Anxiety* and *Fatigue*. Only marginal effects (*dx/dy*) of a change in the regressors on the probability of reporting very good health (*outcome 1*) are commented (Table 4, columns 2 and 4). Looking at Table 4, it seems that marginal effects are quite similar for both population groups. Regarding self-perceived job insecurity, looking at the two population groups, both native-born and migrant workers, the perception of job insecurity decreases the probability of reporting very good health; however, job insecurity reduces the probability of reporting very good health more for migrant workers than for native-born workers. Younger workers have a higher probability of reporting very good health than older workers. Males have a lower probability of reporting very good health than females. Workers with lower education levels have a lower probability of reporting very good health than workers with higher education levels. Managers have a lower probability of reporting very good health than workers who have a nonmanagerial occupational status. Part-time native-born workers have a lower probability of reporting very good health than full-time workers. As the hours spent practicing sports and enjoying cultural or leisure activities outside the home increase, the probability of reporting very good health increases. Workers who have difficulty falling asleep have a lower probability of reporting very good health than workers who do not have such difficulty. Workers who suffered from anxiety and from overall fatigue over the last 12 months have a lower probability of reporting very good health than workers who did not suffered. Regarding countries dummies, results on Austria and on Greece are quite interesting. Compared to UK workers (the UK was considered the reference country), workers from Austria have a lower probability of reporting very good health with migrants showing a lower probability than native-born workers. Compared to UK workers, workers from Greece have a higher probability of reporting very good health with migrants showing a higher probability than native-born workers.

**Table 4:** Marginal effects. *SRH* as four values variable, one *(very good)* four *(bad and very bad)*.

*Very good health*					*Bad* and *very bad health*			
II gen migrants			Natives		II gen migrants		Natives	
*Variable*	*dx/dy*	*P > | z |*	*dx/dy*	*P > | z |*	*dx/dy*	*P > | z |*	*dx/dy*	*P > | z |*
Age1	.23	0.000***	.36	0.000***	-.01	0.000***	-.01	0.000***
Age2	.17	0.000***	.23	0.000***	-.01	0.000***	-.01	0.000***
Age3	.08	0.007***	.14	0.000***	-.00	0.000***	-.00	0.000***
Age4	.01	0.578	.05	0.000***	-.00	0.006***	-.00	0.000***
Male	-.03	0.012**	-.02	0.000***	.00	0.015**	.00	0.000***
Partner	.02	0.128	.00	0.160	-.00	0.143	-.00	0.165
Low Education	-.09	0.000***	-.08	0.000***	.01	0.000***	.00	0.000***
Middle Education	-.06	0.001***	-.03	0.000***	.00	0.002***	.00	0.000***
Manager	-.03	0.079*	-.02	0.001***	.00	0.066*	.00	0.001***
Workedhours	-.00	0.654	-.00	0.399	.00	0.654	.00	0.400
Partime	-.02	0.198	-.01	0.054*	.00	0.225	.00	0.067*
Jobinsecurity	-.05	0.001***	-.03	0.000***	.00	0.006***	.00	0.000***
Sport	.09	0.000***	.08	0.000***	-.00	0.000***	-.00	0.000***
Badsleep	-.13	0.000***	-.11	0.000***	.01	0.000***	.00	0.000***
Anxiety	-.11	0.000***	-.10	0.000***	.01	0.000***	.01	0.000***
Fatigue	-.14	0.000***	-.15	0.000***	.01	0.000***	.01	0.000***
Austria	-.11	0.000***	-.07	0.000***	.01	0.021**	.00	0.001***
Belgium	-.01	0.575	-.07	0.000***	.00	0.594	.00	0.000***
Denmark	.02	0.622	.01	0.334	-.00	0.590	-.00	0.295
Finland	-.09	0.155	-.08	0.000***	.01	0.372	.01	0.000***
France	.01	0.658	-.01	0.304	-.00	0.644	.00	0.337
Germany	-.11	0.000***	-.12	0.000***	.01	0.013**	.01	0.000***
Greece	.37	0.000***	.18	0.000***	-.01	0.000***	-.00	0.000***
Ireland	.10	0.015**	.10	0.000***	-.00	0.001***	-.00	0.000***
Italy	-.14	0.000***	-.15	0.000***	.03	0.032**	.02	0.000
Luxembourg	-.02	0.281	-.08	0.000***	.00	0.322	.01	0.003***
Netherlands	-.05	0.083*	-.07	0.000***	.00	0.173	.00	0.001***
Portugal	-.06	0.181	-.12	0.000***	.00	0.326	.02	0.000***
Spain	-.03	0.188	-.07	0.000***	.00	0.250	.00	0.000***
Sweden	.00	0.957	-.07	0.000***	-.00	0.956	.00	0.001***

*** stat. signif. at 1%;

** stat. signif. at 5%;

* stat. signif. at 10%.

## Discussion

The main weakness of the results is that they provide only a correlation between the dependent variable and the covariates, without identifying the direction of the relationship between self-reported health and the independent variables, especially job insecurity. This implies that the causality link could go in both directions. Therefore, it cannot be said if individuals who report better health work in more stable jobs or working in more secure jobs make them perceive themselves as healthier.

Although the results indicate a correlation, they show that in the EU15, native-born workers have a higher probability of reporting good health than migrant workers. This result is in line with some of the literature (see, for instance, [34, 35]). According to the literature (see, among others, [34]), policies aimed at migrant integration impact the self-reported health of foreign individuals living in European countries. Migrants who live where integration policies are less successful report worse health outcomes, including self-reported health status [35]. The results for Greece are different from those for other countries. In Greece, both native-born and migrant workers have a higher probability of reporting good health compared to workers living in the UK, where migrants reported having a higher probability of reporting good health than native-born workers. Therefore, the ‘healthy migrant effect’, according to which migrant workers are healthier than the overall population in both the sending and the hosting country because of a self-selection bias among immigrants, holds only in Greece. In 2015–2016, Greece registered an increase in refugee arrivals. Until then, Greece had a weak reception and asylum system and dealt with considerable weaknesses in its health and welfare sectors. However, people who received international protection had access to medical care under the same conditions as Greek citizens. Since 2015, overcrowding and poor hygiene have characterized the living conditions of migrants, and it has been a challenge for the Greek health care system to provide adequate health care for refugees [36].

In all other countries, both native-born and migrant workers have a lower probability of reporting good health than in the UK, and the ‘healthy migrant effect’ does not hold. Italy and Austria are the countries where the probability of reporting good health for migrants is the lowest. Austria provides free access to health services to migrants; however, it seems that asylum seekers and undocumented migrants as well as marginalized groups face many obstacles to health services [37]. Italy is traditionally a country of emigration, and this is one of the reasons why Italy seems to only recently to have developed a policy guiding integration efforts; however, Italy has one of the most inclusive models of migrant access to health care [37]. According to the literature (see, among others, [6, 22]), job insecurity is associated with a lower probability of reporting good health. Looking at population groups, the results suggest that migrant workers suffer more from job insecurity than native-born workers. This result can be somewhat aligned with that of Vives et al.’s [38] study that focused on the impact of job insecurity on a different health outcome, i.e., mental health in Spain. The authors found that poor mental health caused by job insecurity differs across social groups and is highest among young female immigrant manual workers. In contrast, Daly et al. [28] found that the impact of insecurity on overall health differs by population groups, with Australian-born workers having worse self-reported health than non-native-born workers. The explanation for this result is that their study focuses on foreign workers (born in India or in the Philippines) who consider precarious work conditions and job insecurity as normal practices, as they come from countries where the labour markets are very informal. Unfortunately, the EWCS6 does not provide information about the nationality of migrant workers. The migrant workers’ lower probability of reporting good health can be explained by the fact that job insecurity is likely to be very stressful for individuals who lack social support [10] and interpersonal relationships, and this seems to be the case for migrant workers more than for native-born workers. Social support is defined as helpful resources coming both from formal/informal interpersonal relationships (originating from family, friends, colleagues), from social networks, and from helpful groups that provide individuals with different kinds of support. Generally, immigrant status is correlated with a lack of social support [39]. This likely happens because of difficulties migrants face in forming interpersonal relationships. For instance, migrants do not always adequately speak the language of the country in which they are working, and this could impose a crucial limitation on their social lives. Indeed, some research shows that migrants receive less social support than native-born individuals [39, 40]. According to Greenhalgh and Rosenblatt [10], social support helps individuals cope with the adversity of everyday life and could lessen the effects of job insecurity on health. Therefore, the migrant workers’ lower probability of reporting good health can be explained by the lack of social support, which might have buffered the impact of the fear of losing their jobs on their health.

Regarding findings for the other covariates, the results for age are in line with those in the literature [41], with older workers having a decreasing probability of reporting good health. In contrast, the results for gender are not in line with those in the literature, with males having a lower probability of reporting good health. As in most of the literature (see, for instance, [42]), the results for education show a positive relationship between education and health. Education has been demonstrated to show that it helps individuals avoid unhealthy behaviours and to gain better access health care. Living with a partner has beneficial effects on health outcomes. This is likely to happen because a partner can provide both social and financial support. This result is also in line with those in the literature. Managers have a lower probability of reporting good health than other workers. This finding could be explained by considering that although managerial status implies a higher compensation, which could have positive effects on health, it requires considerable effort, which, in contrast, could unfavorably impact health. Workers employed part-time report worse health than workers employed full-time. The literature on the relationship between part-time employment and health is mixed. Part-time workers are likely to report worse health when they do not choose this occupational status and would prefer working full-time with the added financial benefit that provides. Physical activity is crucial for preserving good health; indeed, according to the literature, the number of hours workers spend practicing sports and enjoying cultural or leisure activities has a beneficial effect on health both for medical reasons and because such activities imply sharing relationships, which, in turn, benefit health. In line with the literature, sleep problems, including difficulties falling asleep, negatively impact many health outcomes and overall wellbeing.

## Conclusions

The study investigates the correlation between job insecurity and self-reported health (a subjective measure of health) employing data from the Sixth European Working Conditions Survey [43]. Job insecurity refers to self-perceived job insecurity, which is the individual’s subjective evaluation of the possibility of losing their job in the future. The results show that the perception of losing one’s job in the next 6 months decreases the probability of reporting good and very good health more for migrant workers than for native-born workers. Since job insecurity is likely to increase in the future because of the COVID-19 pandemic, which has been causing significant changes in working conditions with workers dealing with new job demands, European institutions should care about this crucial dimension of working conditions and its impact on public health. In addition, public health policies should provide psychological support to help workers better manage stress induced by job insecurity, with the aim of buffering its effects on health outcomes. Another interesting result is that within the EU15, the ‘healthy migrant effect’ holds true only in Greece, with migrant workers reporting worse health than native-born workers. This should stimulate governments to remove barriers to health care use by migrant workers and to extend access to primary health care by providing free health services.
